# Inhibition of Murine Bladder Cancer Cell Growth *In Vitro* by Photocontrollable siRNA Based on Upconversion Fluorescent Nanoparticles

**DOI:** 10.1371/journal.pone.0112713

**Published:** 2014-11-25

**Authors:** Huichen Guo, Dan Yan, Yanquan Wei, Shichong Han, Haisheng Qian, Yunshang Yang, Yingpeng Zhang, Xiangtao Liu, Shiqi Sun

**Affiliations:** 1 State Key Laboratory of Veterinary Etiological Biology and Key Laboratory of Animal Virology of Ministry of Agriculture, Lanzhou Veterinary Research Institute, Chinese Academy of Agricultural Sciences, Xujiaping 1, Lanzhou, Gansu, 730046, The People's Republic of China; 2 School of Medical Engineering, Hefei University of Technology, Hefei 230009, The People's Republic of China; 3 School of Petrochemical Engineering, Lanzhou University of Technology, Lanzhou 730050, The People's Republic of China; Taipei Medical University, Taiwan

## Abstract

This study provides a unique approach to activate caged small interfering RNAs (siRNAs) using indirect UV light emitted by the near-infrared (NIR)-to-UV upconversion process to achieve high spatial and temporal gene interference patterns. siRNA molecules against the anti-apoptotic gene *survivin* was caged by light-sensitive molecules (4,5-dimethoxy-2-nitroacetophenone, DMNPE), which rendered them temporarily non-functional. NIR-to-UV NaYF_4_:Yb,Tm upconversion nanoparticles (UCPs) served as delivery vehicles and activators of the caged siRNA molecules in murine bladder cancer cells (MB49 cell line). Upconverted UV light at 355 nm was emitted from the NIR-irradiated UCPs, which well coincided with the wavelength needed to uncage DMNPE. Consequently, UV light acted as a switch to uncage the delivered siRNA molecule, thereby rendering fully functional for exerting its therapeutic effect in the bladder cancer cells. To achieve the highest RNA interference efficiency, conditions such as time after cellular uptake, excitation time, UCPs concentration and laser power were optimized. Results showed that 200 µg/mL nanoparticle concentration combined with 12 h incubation with MB49 cells and excitation with NIR laser at 100 mW power for 15 min provided the ideal interference efficiency and strongest induction of MB49 cell death. Our findings demonstrate the potential biological application of UCPs in treating bladder cancer by a novel therapeutic approach.

## Introduction

Bladder cancer is the most common malignancy of the genitourinary tract worldwide. Considerable research effort has been devoted to develop new and effective therapies for bladder cancer. In addition to conventional treatment methods such as surgery, chemotherapy and radiotherapy, gene therapy is considered an effective alternative for treatment of tumors.Hence, RNA interference (RNAi), a naturally occurring mechanism in cells for gene silencing, shows strong potential for treatment of bladder cancer [1], [2], [3]. RNAi involves the binding of small interfering RNA (siRNA) to the RNA-induced silencing complex (RISC), which then directs the destruction of messenger RNA (mRNA) that is complementary to the antisense strand of the siRNA. Target-specific RNAi can knock down a gene with high specificity and selectivity, thereby providing an important tool for personalized cancer therapy.

The RNAi machinery is usually initiated as soon as siRNA enters the cell, and the effects on gene silencing can be observed soon afterwards. However, rapid and uncontrolled RNAi limits the utility of gene therapy. Therefore, efforts have been made to develop spatiotemporally inducible RNAi. One promising approach is “caged” RNAi, which utilizes siRNA modified with a photolabile protection group that blocks its activity. Since activity of the “caged” siRNA relies on a light-based trigger (such as UV light), this approach permits spacing, timing, and control over the degree of gene expression, First described by Kaplan in 1978, this method has been used in a variety of applications [4]. In this study, we chose 4,5-dimethoxy-2-nitroacetophenone (DMNPE), a 2-NB(2-nitrobenzyl) class of photolabile protection group, to cage siRNAs for minimal leakiness and maximal uncaging efficiency.

In recent years, upconversion fluorescent nanoparticles (UCPs) have been increasingly used as fluorescent markers and for gene delivery. UCPs show good biocompatibility and no immunogenicity as a gene delivery system, and can be safely excreted without leaving residues in the body. UCPs can effectively deliver siRNA or DNA to target cells or tissues while protecting them from degradation by nucleases in blood serum. In addition, compared with traditional fluorescent markers, such as organic dyes and quantum dots, UCPs exhibit low toxicity, good chemical stability, high fluorescence intensity, high stability, and large Stokes shift when used as fluorescent markers. UCPs are excited by near-infrared (NIR) light, which is affected minimally by interference and scatter of auto-fluorescence from tissues and cells, and thus offer low background and high signal-to-noise ratio [5].

NIR light can penetrate tissues deeper than visible light, and as such, UCPs can be used as fluorescent markers or in optical treatment of deep tissues. Hence, UCPs exhibit good prospect as fluorescent markers and gene delivery vectors in clinical detection, treatment, and analysis of biological molecules [6], [7], [8], [9]. NaYF4:Yb,Er/Tm exhibits the highest efficiency from the NIR to visible/upconversion fluorescence and can provide wavelengths from ultraviolet light to infrared light. Therefore, NaYF4:Yb,Tm was used as a photocaged siRNA carrier in this study.

*Survivin* is one of the strongest inhibitors of proteins involved in apoptosis. Given the large difference in its expression between normal and malignant tissues and its causal role in cancer progression, survivin has been studied as a target for anti-cancer therapy and as a tumor marker [10], [11], [12], [13]. Here, survivin was chosen as the target gene in order to investigate the effectiveness of RNAi-based gene therapy in treating bladder cancer. Specifically, survivin siRNA caged with DMNPE was combined with UCPs as the carrier. Activation of siRNA was achieved by irradiation with 980 nm NIR laser and inhibition of bladder cancer cell growth was assessed. Our results provide new insights into the use of UCPs in gene therapy.

## Materials and Methods

### 1. Cell

MB49-PSA cells which were provided by Dr. Ratha Mahendran from Faculty of Medicine (NUS, Singapore) [7], [9], were cultured at 37°C under a 5% CO_2_ atmosphere in modified Eagle's medium (MEM, Gibco) supplemented with 10% fetal bovine serum (Gibco) containing 100 units/mL penicillin G sodium (BBI) and 100 µg/mL streptomycin sulfate (BBI).

### 2. Amino group-modified UCPs and UCPs/siRNA-DMNPE complex formation

NaYF_4_:Yb,Tm nanocrystals and monodisperse silica-coated NaYF_4_:Yb,Tm (NaYF4:Yb,Tm@silica, UCPs) were synthesized and characterized following the method described by Guo et al. (2010) [7] and Qian et al. (2009) [9]. The UCPs were modified using an amino group following the method described by Guo et al. (2011). DMNPE (Invitrogen) was used to cage siRNAs following the manufacturer's protocol, and UCPs/siRNA-DMNPE complexes were made as described previously by Guo et al. (2011) [14]. The ratio of UCPs to siRNA was determined by measuring the zeta potential using Nano-ZS ZEN 3600 (Malvern Instruments Ltd, UK) at 25°C. The UV absorption spectrum was gathered using BioMate 3S UV-visible spectrophotometer (Thermo Scientific) at 25°C.

### 3. Cellular uptake of UCPs at different time points

MB49 cells were seeded on 6 well plates and cultured for 24 h. The cells were immediately treated with nanoparticles at 100 µg/mL and collected at different time points (15 min, 1 h, 6 h, and 24 h). Cells loaded with nanoparticles were fixed in 4% paraformaldehyde for 10 min at room temperature (RT) and then washed twice with 1× PBS for 5 min. The nuclei were counterstained with 0.1 µg/mL DAPI (Sigma) for 5 min at RT followed by two washes with 1× PBS for 5 min each. Nanoparticles loaded on the fixed cells were then visualized under a confocal laser scanning microscope (Nikon C1 Confocal) fitted with a continuous wave 980 nm laser excitation source.

### 4. Quantification of UCPs uptake

Cells were seeded into 75 cm^2^ flasks at 1×10^6^/mL after culturing for 24 h. The cells were immediately treated with nanoparticles at 100 µg/mL and collected at different times point at 0, 15 min, 1 h, 3 h, 6 h, 24 h, 48 h, and 72 h. Intracellular uptake of the nanoparticles was assessed by measuring total amount of nanoparticles in cells using Inductively Coupled Plasma Optical Emission Spectrometer (ICP-AES).

### 5. Transfection of cultured cells

MB49-PSA cells were transfected with the UCPs/siRNA-DMNPE complex following the method described by Guo et al. (2011) [14]. Briefly, UCPs/siRNA-DMNPE complex was incubated with cells for different time point as indicated. at 37°C under 5% CO_2_ atmosphere. At the end of the incubation period, the cells were excited with or without 980 nm laser using a 980 nm VA-II diode pumped solid state (DPSS) laser source.

### 6. *In vitro* viability/cytotoxicity tests

Cell viability was assessed using Cell Titer 96 aqueous one solution assay (Promega, Madison, WI). MB49 cells were seeded onto a 96-well plate at a density of 5×10^3^ cells per well. After one day in culture, UCPs/siRNA-DMNPE complex was added to the wells. The cells were incubated for different time point as indicated and then excited using 980 nm laser or not. After incubation for an additional 24 h at 37°C, 20 µl of the reagent was directly added to the wells and incubated for another 24 h. Quantity of the formazan product formed as indicated by 490 nm absorbance is directly proportional to the number of living cells in the culture. Each experiment was done in triplicate. Cell viability (%) relative to the control wells containing cell culture medium without nanocrystals was calculated as [*A*]_expt_/[*A*]_control_×100%, where [*A*]_expt_ is the absorbance of the test sample and [I]_control_ is the absorbance of the control sample.

### 7. Detection of survivin expression by immunoblot analysis

MB49 cells were seeded onto a six-well plate and cultured until the confluence was 80% to 90% (which occurred after approximately 24 h). At that point, UCPs/siRNA-DMNPE complex was added and the cells were incubated for different time point as indicated. Then, the cells were irradiated with or without 980 nm laser and incubated at 37°C for another 24 hours. The cells were collected using cold cell lysis reagent (Pierce) as per the manufacturer's protocol. Proteins in cell lysates were separated by SDS-PAGE on 12% acrylamide gels using a discontinuous buffer system. They were then transferred to a nitrocellulose membrane (Pierce) in transfer buffer (20 mM Tris-HCl, 190 mM glycine, 20% methanol, pH 8.3) using a Mini Trans-blot transfer system (Bio-Rad) at 100 V for 1 h. The membranes were blocked with 5% non-fat dried milk in Tris-buffered saline containing 0.05% Tween-20 (TBST) at RT for 1 h and then incubated with anti-survivin antibody (Santa Cruz, 1∶200 dilution) at RT for 1 h. After three washes in TBST, the membranes were incubated with peroxidase-conjugated anti-mouse secondary antibody (Sigma, 1∶8000 dilution) at RT for another 1 h. Three additional washes in TBST were performed before the NC membrane were visualized with ECL solution (Pierce).

### 8. Statistical analysis

The films were scanned as gray scale 8-bit images and the density of bands were determined by the ImageJ software.The data of relative quantity in western blot are presented and the statistical analysis of variance between groups was performed by SPSS Statistics 19.0 software. Significant difference of all statistical tests was set at 0.05 (p<0.05).

## Results

**Figure 1:**
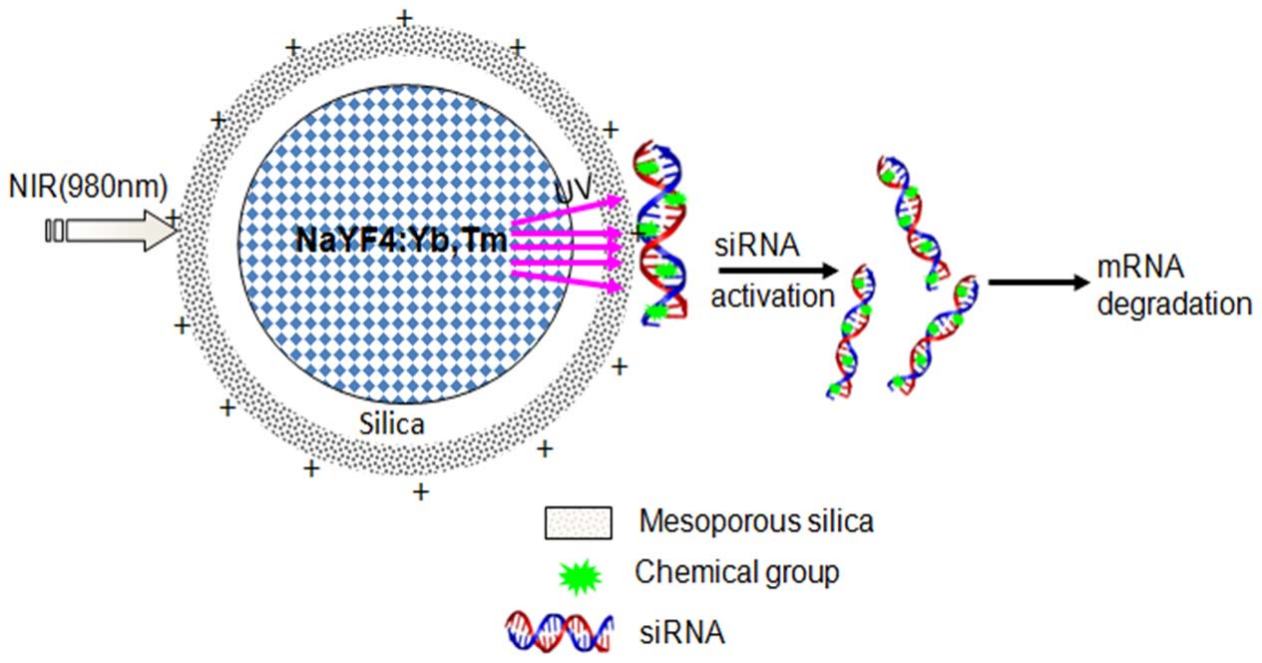
Schematic diagram of UCPs as carriers and light source of caged siRNA.

**Figure 2:**
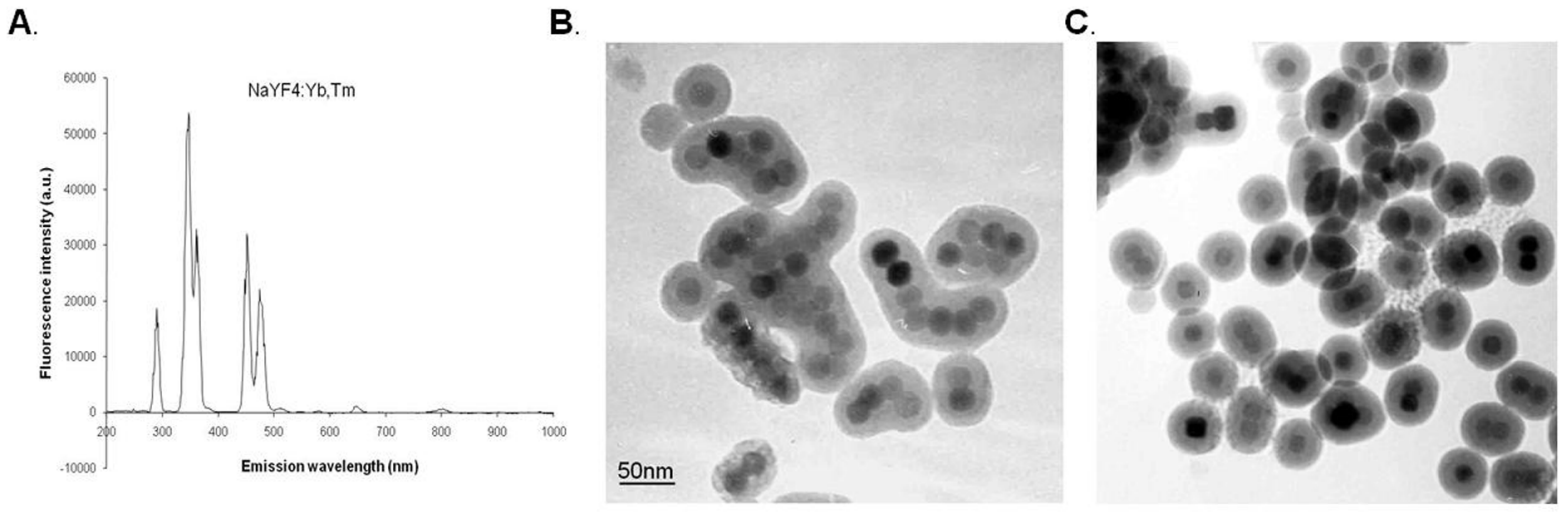
Physicochemical characterization of nanoparticles. Fluorescence spectra of unmodified nanoparticles (A); morphology of unmodified nanoparticles (B) and amino-functionalized nanoparticles (C) observed by TEM.

In this study, NaYF_4_:25%Yb,0.3%Tm NIR-to-UV UCPs was used as carrier of caged siRNAs, which were activated using NIR-to-UV upconverted light (**Fig. 1**). As shown in **Figure 2B**, the synthesized nanoparticles possessed a core-shell structure composed of a NaYF_4_ nanocrystal core and a silica shell. To attach the caged siRNAs, the UCPs were modified with an amino group which make its surface is positive charge. The modified nanoparticles shown in **Figure 2C** are more dispersed than those in **Figure 2B**, which can be due to electrostatic repulsion caused by positive charges on the nanoparticle surface. Overall, the nanoparticles were found to be well-dispersed in water, forming a clear colloidal solution and exhibiting strong upconversion fluorescence (**Fig. 2A**).

**Figure 3:**
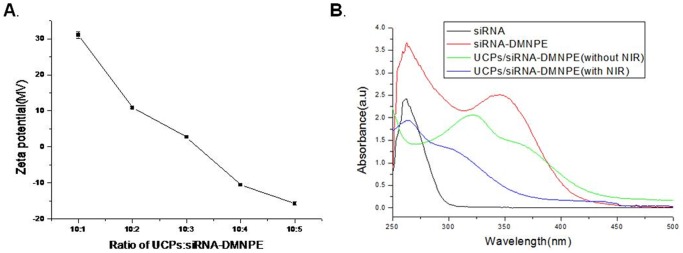
Intracellular uptake study of nanoparticles loaded into MB49 cell lines. (A) Confocal imaging of upconversion fluorescence emitted by MB49 cells loaded with 100 µg/mL of the nanoparticles. Excitation with 980 nm NIR laser emitted green and red visible fluorescence. Nuclei were counterstained with DAPI (blue fluorescence). All of the images were obtained under the same settings, which permitted calculation of relative fluorescence intensity of differentially loaded cells. (B) Intracellular uptake of nanoparticles in loaded cells by ICP-AES for yttrium content.

Complex formation of the amino-modified UCPs with siRNA was examined by measuring the nanozeta potential. UCPs and siRNA were mixed for 1 h at 4°C. The UCPs/siRNA complexes were washed several times with PBS and then resuspended in PBS buffer. Measurement of zeta potential demonstrated that the positive charge on UCPs was gradually neutralized by the negative charge of siRNA with increase in nanoparticle/siRNA ratio (**Fig. 3A**). However, the surfaces of cells are negatively charged in neutral condition (pH 7.0). Hence, to maximize the capacity of UCPs to deliver as much siRNA as possible and to obtain optimal cellular uptake efficiency of the UCPs/siRNA complex, we used a nanoparticle∶siRNA ratio of 10∶2 (mol∶mol) in all *in vitro* experiments. In order to evaluate the efficiency of siRNA uncaging by UCPs, their absorbance was measured. As shown in Figure 3B, DMNPE-caged siRNA revealed a characteristic summit at 355 nm as DMNPE and as summit at 260 nm as siRNA, which demonstrated the attachment of DMNPE groups to the siRNA molecules. For DMNPE-caged siRNA molecules,without irradiation with a 980 nm NIR laser, showed a confused spectra. It estimated that there is interference between the emission of UCPs and that of DMNPE. However, UCPs/siRNA-DMNPE, a significant drop in the 355 nm peak was observed upon irradiation with NIR light.Hence, it is believable that NIR-to-UV UCNs can uncage siRNA molecules under excitation of NIR light.

**Figure 4:**
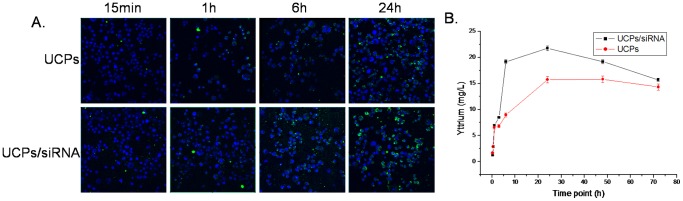
Studies on complex formation of amino-modified nanoparticles with siRNA-DMNPE. (A) Zeta potential of UCPs/siRNA-DMNPE complex at mol/mol ratios of 10∶1, 10∶2, 10∶3, 10∶4, and 10∶5; (B) Absorbance spectrophotometry of siRNA, DMNPE-caged siRNA without irradiation, and UCPs/siRNA-DMNPE activated with or without NIR irradiation.

To determine the level of cellular uptake of UCPs, UCPs/siRNA or UCPs were passively loaded into MB49 cells by incubating them with 100 µg/mL nanoparticles for different durations. As shown in **Figure 4A**, two filters were used to capture green and blue fluorescence emissions. The nuclei were counterstained with DAPI to demonstrate that virtually all of the cells were loaded. A progressive increase was observed in the upconversion fluorescence emitted by nanoparticle-loaded cells with time. However, the fluorescent intensity of UCPs/siRNA complex in MB49 cells was stronger than that of UCPs alone after 6 h incubation. This indicated that MB49 cells had taken up UCPs/siRNA complexes more strongly than UCPs with time increase, which may be due to the surface charge of the nanoparticles. Furthermore, we measured the intracellular uptake of nanoparticles in loaded cells by ICP-AES for yttrium concentration and observed a similar trend of increase in nanoparticle uptake into cells over time (**Fig. 4B**). However, the quantity of UCPs in cells reached the peak and then remained the balance.

**Figure 5:**
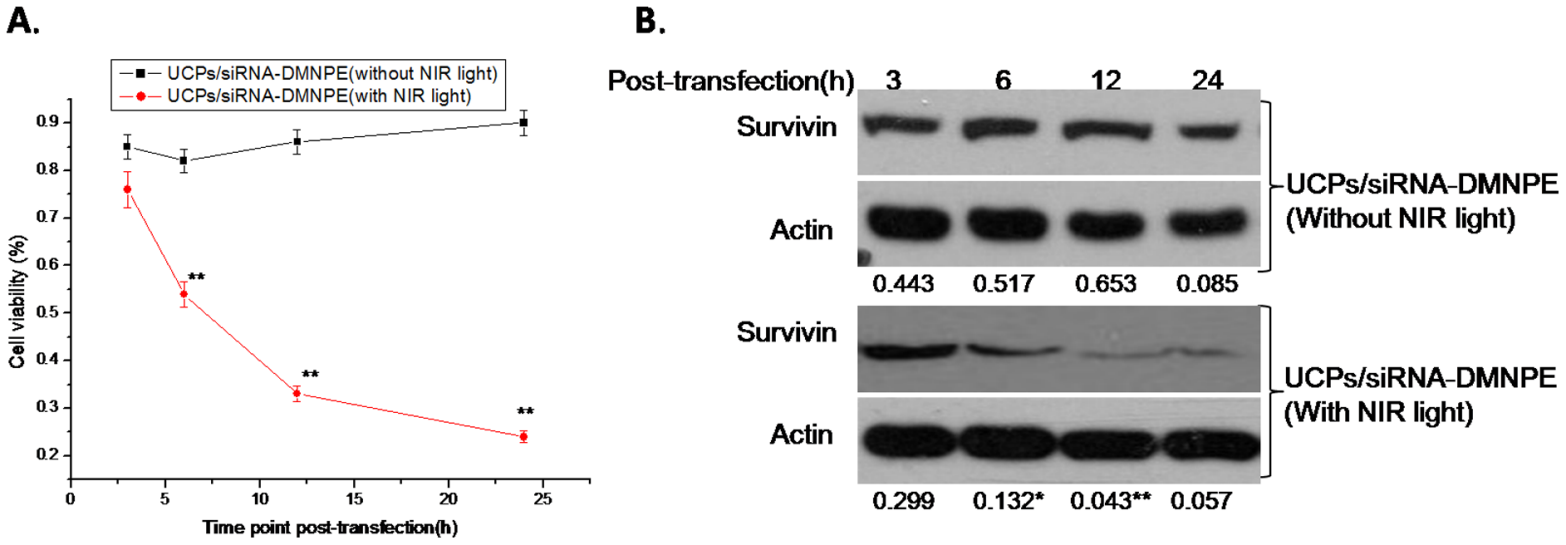
Interference efficiency of UCPs/siRNA-DMNPE at different time points after transfection. UCPs/siRNA-DMNPE was added to MB49 cells at UCPs concentration of 100 µg/mL. The cells were excited using NIR laser light (100 mW) for 15 min at different time points. The cells were collected at 24 h after light treatment. RNAi interference efficiency was determined by immunoblot (A) and MTT assay (B). The experiment was repeated three times with similar results and the result of one representative experiment is shown. Asterisks indicate significant differences between the samples with different treatment at the same time point. (*P<0.05,** P<0.01).

**Figure 6:**
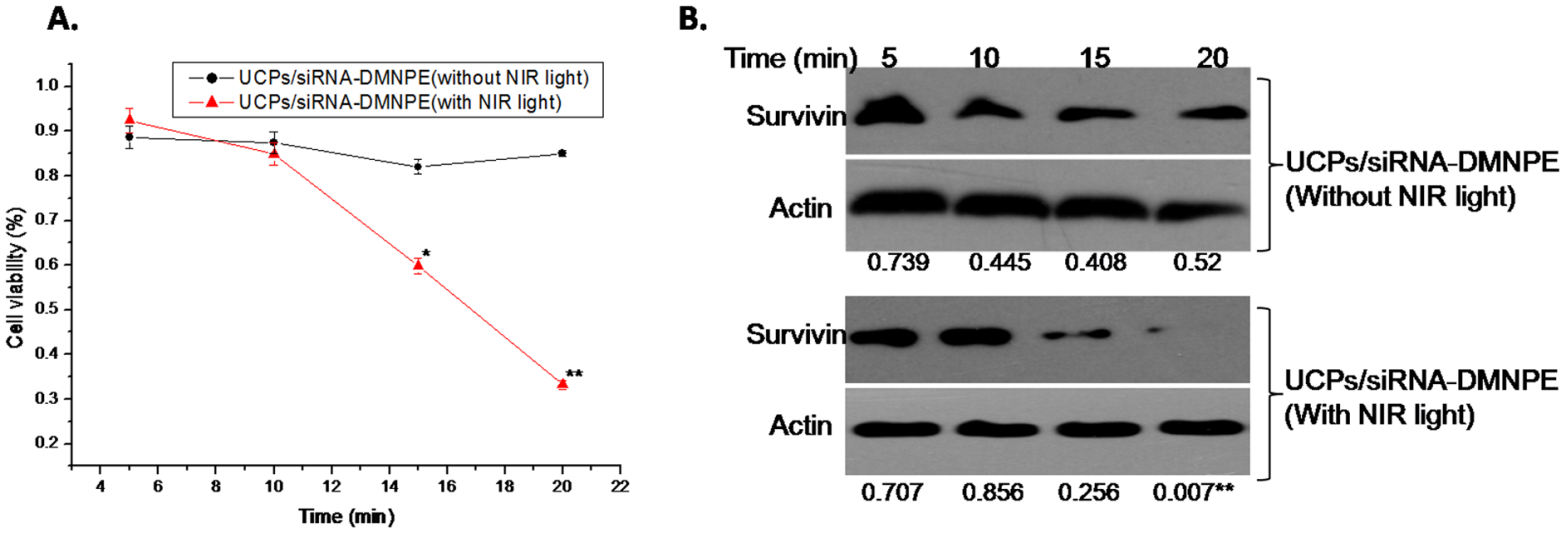
Interference efficiency of UCPs/siRNA-DMNPE with different excitation times. UCPs/siRNA-DMNPE was added to MB49 cells at UCPs concentration of 100 µg/mL. The cells were excited using NIR laser light (100 mW) with different excitation times. The cells were collected at 24 h after light treatment. RNAi efficiency was determined by immunoblot (A) and MTT assay (B). The experiment was repeated three times with similar results and the result of one representative experiment is shown. Asterisks indicate significant differences between the samples with similar excitation times (*P<0.05,** P<0.01).

**Figure 7:**
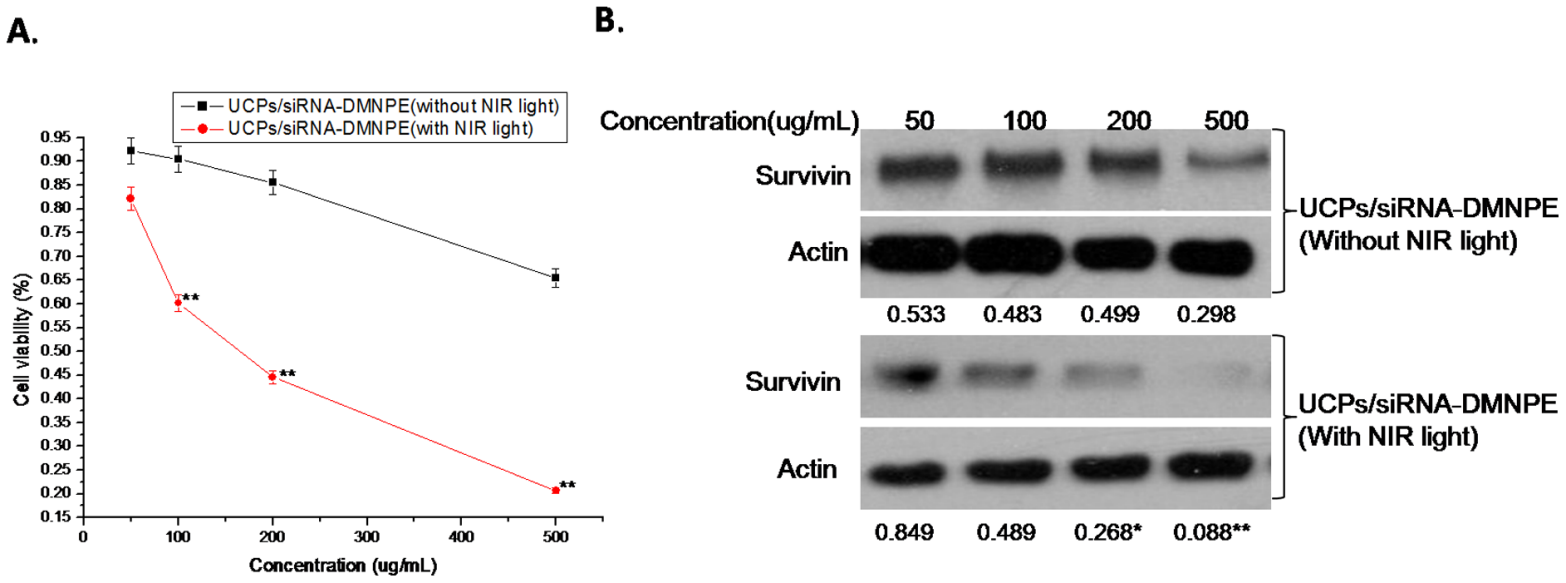
Interference efficiency of UCPs/siRNA-DMNPE at different concentrations. Different concentrations of UCPs/siRNA-DMNPE were added to MB49 cells for 12 h. The cells were excited by NIR laser light (100 mw) for 15 min. The cells were collected at 24 h after light treatment. RNAi efficiency was determined by immunoblot (A) and MTT assay (B). The experiment was repeated three times with similar results and the result of one representative experiment is shown. Asterisks indicate significant differences between the samples with similar concentration (*P<0.05,** P<0.01).

**Figure 8:**
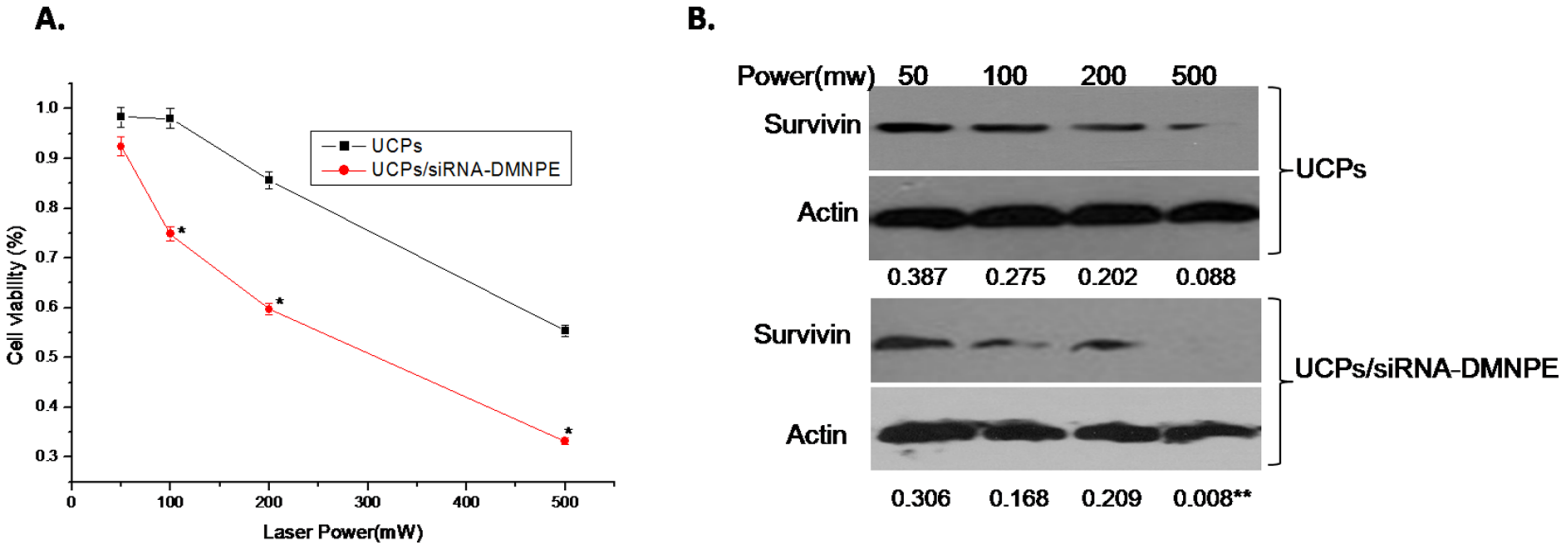
Interference efficiency of UCPs/siRNA-DMNPE at different laser power. UCPs/siRNA-DMNPE was added to MB49 cells at UCPs concentration of 100 µg/mL. The cells were excited using NIR laser light with different power for 15 min. The cells were collected at 24 h after light treatment. RNAi efficiency was determined by immunoblot (A) and MTT assay (B). The experiment was repeated three times with similar results and the result of one representative experiment is shown. Asterisks indicate significant differences between the samples with similar laser power (*P<0.05,** P<0.01).

To determine the optimal conditions for siRNA interference after NIR-laser excitation, experiments were set up with different conditions of time points after transfection, excitation times, concentration of complexes, and laser power. As shown in **Figure 5**, the interference efficiency increased with duration of incubation of nanoparticles with the cells. The cell viability decreased significantly in UCPs/siRNA-DMNPE treatment after excitation with NIR ligh. However, there was almost no change in cell viability without NIR excitation(Fig. 5A).Similarly, at 12 h after treatment with or without NIR excitation, the expression level of suvivin protein decreased significantly and almost remained at the same level at 24 h, which suggested increase in interference efficiency(Fig. 5B).This may be due to increase in cellular uptake of nanoparticles with time, and is consistent with the results shown in **Figure 4**. As shown in **Figure 6**, the interference efficiency increased with time of excitation. In particular, survivin expression decreased significantly when the cells were excited by NIR light for more than 15 min. **Figure 7** shows that the interference efficiency increased with increase in concentration of nanoparticles, and thus the interference efficiency also increased with the quantity of siRNA against survivin. To determine the influence of laser power on interference efficiency, the cells were incubated with UCPs or UCPs/siRNA-DMNPE, which is different from those used in the previous experiment. As shown in **Figure 8**, the interference efficiency also increased with increase in laser power, particularly at 500 mW. However, survivin expression also decreased in the UCPs control group at 500 mW, which suggested that UCPs can induce cell damage once excited by NIR light at high power. Hence, on the basis of the above results, the optimal conditions (12 h incubation, 15 min excitation, 200 µg/mL nanoparticle concentration, and 100 mW laser power) were used to verify the maximal interference efficiency.

**Figure 9:**
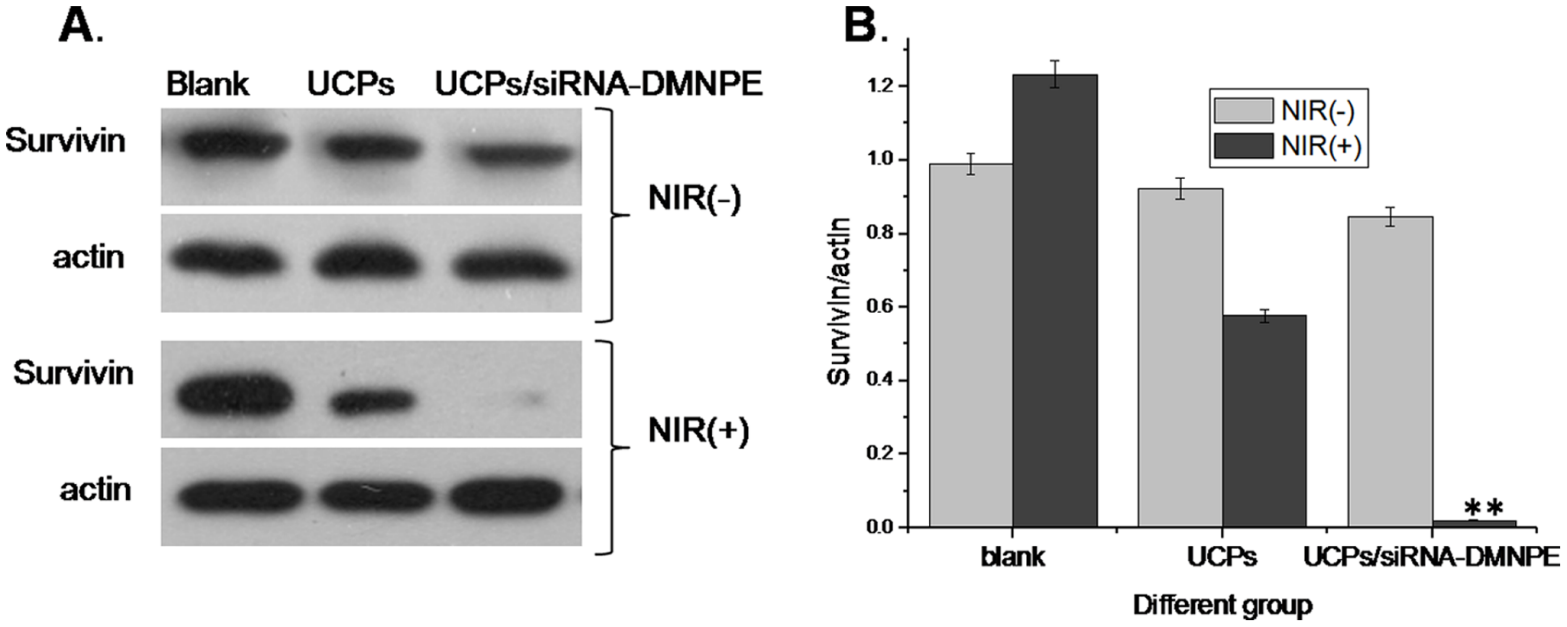
Confirmation of optimal conditions for achieving maximal interference efficiency using UCPs/siRNA-DMNPE. UCPs/siRNA-DMNPE was added to MB49 cells at UCPs concentration of 100 µg/µL. The cells were excited using NIR laser light (100 mW) for 15 min at 12 h after incubation. The cells were collected at 24 h after light treatment. RNAi efficiency was determined by immunoblot (A). Levels of survivin protein relative to those of actin with or without NIR-induced excitation are shown. The experiment was repeated three times with similar results and the result of one representative experiment is shown. Asterisks indicate significant differences between the indicated samples (*P<0.05,** P<0.01).

To confirm whether all of the conditions are optimal, the siRNA interference experiment was performed using UCPs and UCPs/siRNA-DMNPE complex. As shown in the **Figure 9**, almost no decrease in survivin protein was found in the blank control group after excitation with or without NIR light.Howeve, the interference efficiency of UCPs/siRNA-DMNPE was high after excitation with NIR light. In addition, the UCPs also affected expression of survivin to some extent, which may be due to several factors related to lasers and nanoparticles.

## Discussion

Gene regulation is important in living systems because gene expression levels play important roles in gene functions. Methods for controlling gene expression (up- or down-regulation) revolutionized the field of developmental biology and gene therapy. The recent development of photocontrollable gene expression holds great promise for increased spatiotemporal control over modulation of gene expression. In this technique, transcriptional activators/plasmid for gene expression or siRNAs for RNAi are caged with light-sensitive molecules that render them temporarily non-functional. This process, known as photocaging, can be done in a variety of ways, the most common of which is modifying select base pairs or chemical bonds in the caging molecule. Upon irradiation with UV light, the modification is destroyed, thereby re-activating the nucleic acids to regain their function [15], [16], [17]. In this way, very high spatial and temporal control over gene expression patterns can be achieved. However, most of the current photoactive systems are only suitable for *in vitro* applications because UV light, which is very harmful, is necessary for the uncaging process. UV light also exhibits poor tissue penetration depth, so it is unacceptable for *in vivo* and clinical use. In addition, although the use of siRNA for RNAi shows great potential for therapy, siRNA is easily degraded by RNase in blood serum and are hence unsuitable for *in vivo* use. siRNA nucleotides are relatively small and short, even after chemical modification to enhance siRNA stability. These nucleotides can be excreted through the urinary tract after absorbance in the blood. siRNA also needs to overcome endothelial barriers to reach the target cells in different organisms. Hence, developing a system for efficient and safe delivery of siRNA to cells is highly important. To date, delivery systems for siRNA have included nonvirus- and virus-based delivery systems; the former includes liposomes, nanoparticles, cationic polymers, micelles and other systems based on these forms. Given that virus-based delivery systems exhibit some side effects including potential immunogenicity and tumorigenicity in gene therapy, they are unsuitable for siRNA delivery. Hence, at present, nonvirus-based delivery systems for siRNA are being explored. In this study, NaYF_4_:25%Yb,0.3%Tm NIR-to-UV UCPs were used as carriers of caged siRNA. NIR light, which was used to excite UCPs, can penetrate biological tissue more than visible or UV light, thereby enabling the use of UCPs as carriers in deep tissues.

Photolabile protection groups can be divided into two kinds: the unspecific caging group involves random caging of the siRNA phosphate backbone [18], while the other group involves specific blocking of the 5′-phosphate of siRNAs [19]. The nonspecific groups are more stable than the specific groups. In addition, the nonspecific groups are easy to synthesize by chemical or biochemical methods, thereby reducing the cost of producing caged siRNA. In this study, siRNA was caged using DMNPE. DMNPE belongs to the 2-NB caging group and its derivatives are the most widely utilized and well characterized caging molecules [20]. The NIR-to-UV UCPs spectrum shows an emission peak at 350 nm, which coincides well with the wavelength needed to uncage DMNPE. In addition, NIR light is only weakly absorbed by biological tissues, which ensures maximal NIR light absorbance of UCPs and increases the sensitivity of the method.

To verify the optimal time point of NIR excitation in cells, the cellular uptake of UCPs was detected by fluorescent microscopy and ICP-AES. The quantity of UCPs in the cells increased initially with time but decreased after 24 h post inoculation. This indicates that the early stage of incubation with UCPs is critical. Considering cell viability, we selected 12 h post-incubation of UCPs for NIR-induced excitation. Results of our subsequent experiments confirmed that this time point is ideal to achieve high RNA interference. Although NIR light is less cytotoxic than UV light, use of high laser power and long excitation time can induce significant cytotoxicity and lower RNAi efficiency. Hence, only 15 min of excitation time was used and the laser power was fixed to 100 mW. In addition, since UCPs induce damage to cells at high concentrations, the concentration of UCPs was set at 200 µg/mL. The high RNAi efficiency observed in the study validated the suitability of these parameters for our experiments.

## Conclusions

In conclusion, this study demonstrates that NIR-to-UV UCPs can serve as delivery vehicles and activators of caged nucleic acids. Irradiation of UCPs in cells by upconverted UV light emitted from NIR led to uncaging of the loaded molecules, thereby rendering them fully functional in host cells. In addition to exhibiting good light penetration in tissues for in-depth photoactivation, NIR light-induced excitation needed for the NIR-to-UV upconversion process caused minimal photodamage to biological specimens. The intrinsic fluorescence properties of these nanoparticles enabled emission that led to release of caged siRNA and facilitation of gene interference. Thus, UCPs can provide a platform for applications involving remote control of RNAi and gene therapy of cancer. Furthermore, our findings demonstrate the utility of multicolor upconversion luminescence UCPs as a versatile and powerful tool for advanced applications in imaging, biodetection, and gene therapy.
